# Abnormally glymphatic system functional in patients with migraine: a diffusion kurtosis imaging study

**DOI:** 10.1186/s10194-024-01825-z

**Published:** 2024-07-22

**Authors:** Yungang Cao, Mei Huang, Fangwang Fu, Keyang Chen, Kun Liu, Jinming Cheng, Yan Li, Xiaozheng Liu

**Affiliations:** 1 Department of Neurology of the Second Affiliated Hospital, Yuying Children’s Hospital, Wenzhou Medical University, Wenzhou, Zhejiang 325027 China; 2 Department of Radiology of the Second Affiliated Hospital, Yuying Children’s Hospital, Wenzhou Medical University, Wenzhou, Zhejiang 325027 China; 3 Department of Neurology of the Hebei General Hospital, Shijiazhuang, Hebei 050051 China; 4 Wenzhou Key Laboratory of Structural and Functional Imaging, Wenzhou, Zhejiang 325027 China

**Keywords:** Migraine, Diffusion tensor imaging analysis along the perivascular space, Diffusion kurtosis imaging analysis along the perivascular space, Glymphatic system

## Abstract

**Background:**

The diffusion tensor imaging analysis along the perivascular space (DTI-ALPS) method has been used to evaluate glymphatic system function in patients with migraine. However, since the diffusion tensor model cannot accurately describe the diffusion coefficient of the nerve fibre crossing region, we proposed a diffusion kurtosis imaging ALPS (DKI-ALPS) method to evaluate glymphatic system function in patients with migraine.

**Methods:**

The study included 29 healthy controls and 37 patients with migraine. We used diffusion imaging data from a 3T MRI scanner to calculate DTI-ALPS and DKI-ALPS indices of the two groups. We compared the DTI-ALPS and DKI-ALPS indices between the two groups using a two-sample t-test and performed correlation analyses with clinical variables.

**Results:**

There was no significant difference in DTI-ALPS index between the two groups. Patients with migraine showed a significantly increased right DKI-ALPS index compared to healthy controls (1.6858 vs. 1.5729; *p* = 0.0301). There was no significant correlation between ALPS indices and clinical variables.

**Conclusions:**

DKI-ALPS is a potential method to assess glymphatic system function and patients with migraine do not have impaired glymphatic system function.

## Introduction

Migraine is a common chronic disorder of the brain that has become a major public health problem worldwide. Migraine has a high incidence and a long disease course, ranking second in the burden of neurological diseases. Approximately 1.04 billion people worldwide suffer from migraine, with a lifetime prevalence of approximately 10% in men and 22% in women [1]. However, the pathological mechanism of migraine remains unclear.

Recent research has suggested that the glymphatic system, a “waste removal” system in the central nervous system, may be involved in the pathogenesis of migraine [2]. Mice in the migraine model showed decreased glymphatic influx with reduced aquaporin-4 expression and impaired polarisation, suggesting glymphatic dysfunction in the migraine mouse model [3]. Acupuncture-induced cortical spreading depression (CSD) in the cerebral cortex of mice resulted in delayed and slowed glymphatic flow [4]. This phenomenon also seemed to explain the delayed onset of pain in patients with migraine with aura [4].

In functional magnetic resonance imaging (fMRI) studies, diffusion tensor imaging analysis along the perivascular space (DTI-ALPS) method has been used to evaluate the function of the glymphatic system in patients with migraine [5, 6]. Zhang et al. found that the DTI-ALPS index in patients with chronic migraine (CM) was higher than that of healthy controls (HC) and patients with episodic migraine; they believed that in the process of migraine chronicity, the activity of the glymphatic system increases [5]. However, another study in patients with migraine found no difference in the DTI-ALPS index between patients with migraine and healthy controls [6].

Although DTI-ALPS has been a widely used method for describing glymphatic system function. However, due to the shortcomings of the diffusion tensor model in accurately describing the diffusion information of the nerve fibre crossing region, the DTI-ALPS index of glymphatic system function may be inaccurate. Methods based on high angle resolution diffusion imaging data and constrained spherical deconvolution have shown that 90% of the white matter of the brain has crossing fibres, including projection fibres and association fibres [7].

Diffusion kurtosis imaging (DKI) is a popular high-order diffusion imaging method that uses a fourth-order statistical model to describe the diffusion of water molecules to solve the problem of nerve fibre crossing, and has been widely used in the study of clinical white matter microstructure [8, 9]. In the current study, we proposed a diffusion kurtosis imaging ALPS (DKI-ALPS) method to evaluate the function of the glymphatic system in patients with migraine. We believe that the DKI-ALPS method could be used as a potential indicator of the glymphatic system.

## Methods

### Participants

**Fig. 1:**
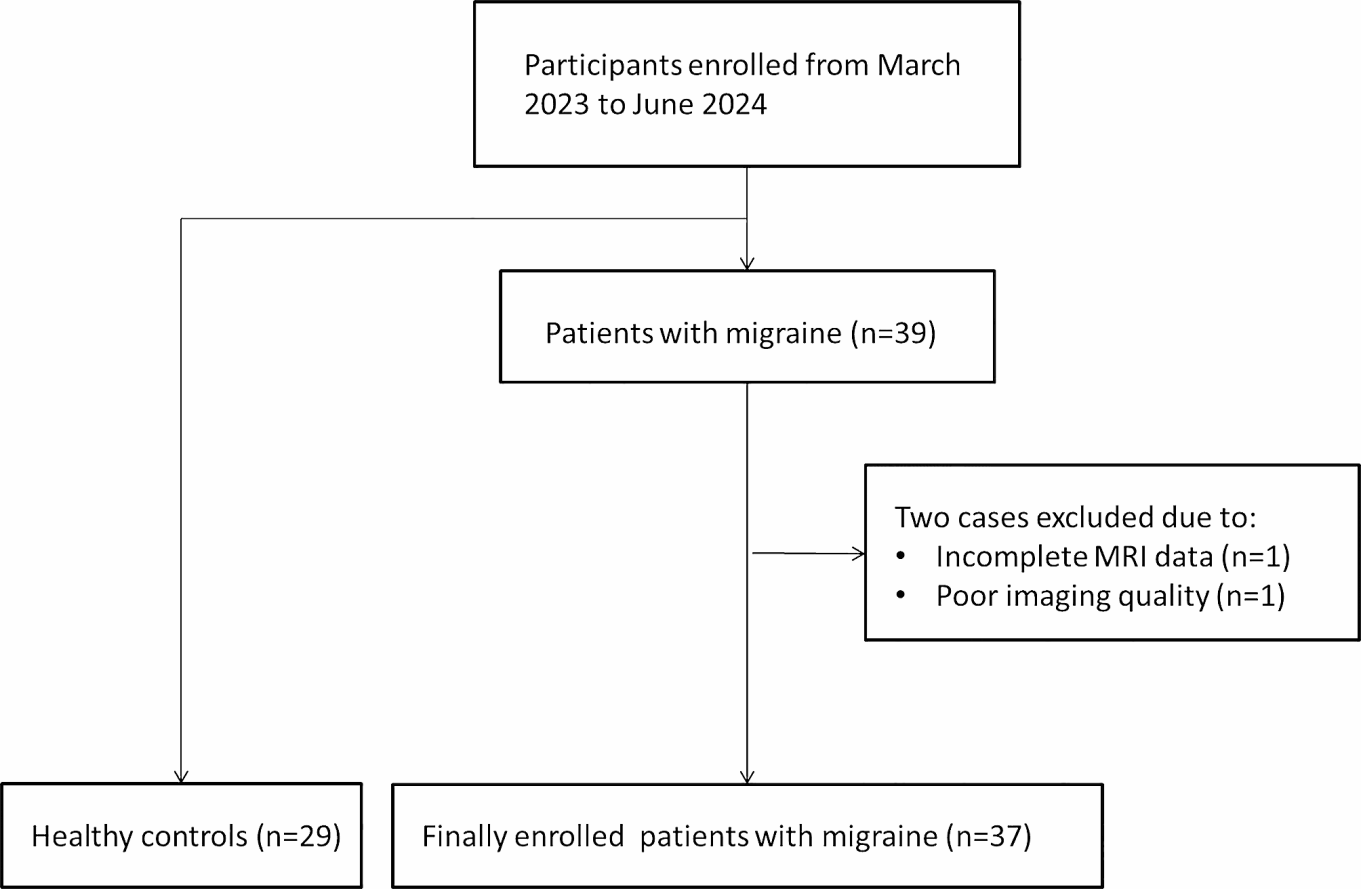
Flowchart of participant enrollment

39 patients with migraine and 29 age- and sex-matched healthy controls were enrolled in the study, which took place at a tertiary headache center, between March 2023 and June 2024 (Fig. 1). Two neurologists who specialize in headache disorders assessed MRI and neuropsychological findings and made a definitive migraine diagnosis following the criteria of the International Headache Association (International Classification Committee for Headache and Headache, 2018) [10]. The following criteria must be met by patients: (a) they must be right-handed; (b) they must be at least eighteen years old; (c) they must have had migraine symptoms for more than six months; (d) they must have at least two headache attacks per month (as attested to by the patient’s pre-study self-report); and (e) they must not have experienced a headache attack or take acute migraine medication for at least three days before the fMRI scan. Headache attacks that occurred three days before, on the day of, or following the scan; a history of substance abuse or preventative drug use; mental health conditions; MRI scanning contraindications; and female subjects who are menstruating or pregnant were among the exclusion criteria. The local community was used to find control subjects, who had no prior history of neurological conditions. They do not take any medication and do not experience headaches or migraines. The Institutional Review Board of our university approved this study. Before surgery, all participants provided written, informed consent.

### Neurophysiological assessment

Demographic data included age, sex, and the following clinical characteristics obtained from patients with migraine: disease duration, attack frequency, and relevant psychological tests. The Headache Impact Test-6 (HIT-6) and the Migraine Disability Assessment (MIDAS) were used to assess the impact of migraine [11, 12]. The Patient Health Questionnaire-9 (PHQ-9) and the Generalised Anxiety Disorder-7 (GAD-7) were used to assess depression and anxiety, respectively [13, 14].

### MRI scan

Images were acquired on a 3.0 Tesla magnetic resonance imaging scanner (Ingenia Elition, Philips Medical, Best, the Netherlands). The MRI protocol included coronal T2-weighted imaging, 3D T1-weighted imaging, and diffusion MRI. T2-weighed imaging was used to evaluate the structural abnormalities of the brain. Diffusion MRI data were acquired using a single-shot EPI sequence with a multishell scheme using the following parameters: repetition time = 3109 ms, echo time = 89 ms, acquisition matrix = 112 × 112, field of view = 224 × 224 mm^2^, slice thickness = 2 mm, flip angle = 90°, diffusion sensitivity coefficient (b) = 1000, 2000 s/mm^2^ with 56 encoding diffusion directions per each *b*-shell, and one b = 0 s/mm^2^ scan (b0), multiband factor = 3; parallel imaging factor = 1.3. The acquisition time was 6 min. We instructed patients to remain still and close their eyes during image acquisition.

### Data processing

Diffusion MRI data were pre-processed using FSL6.0.6 (https://fsl.fmrib.ox.ac.uk/fsl/) and mrtrix3 (https://www.mrtrix.org/). The preprocessing steps include skull stripping, eddy current and head motion correction, PCA denoising and Gibbs ringing artefact removal. Based on the pre-processed diffusion MRI data, we used the Diffusional Kurtosis Estimator (DKE) to estimate both the diffusion tensor and kurtosis tensor [15].

### Diffusion kurtosis image analysis along with the perivascular index calculation

**Fig. 2:**
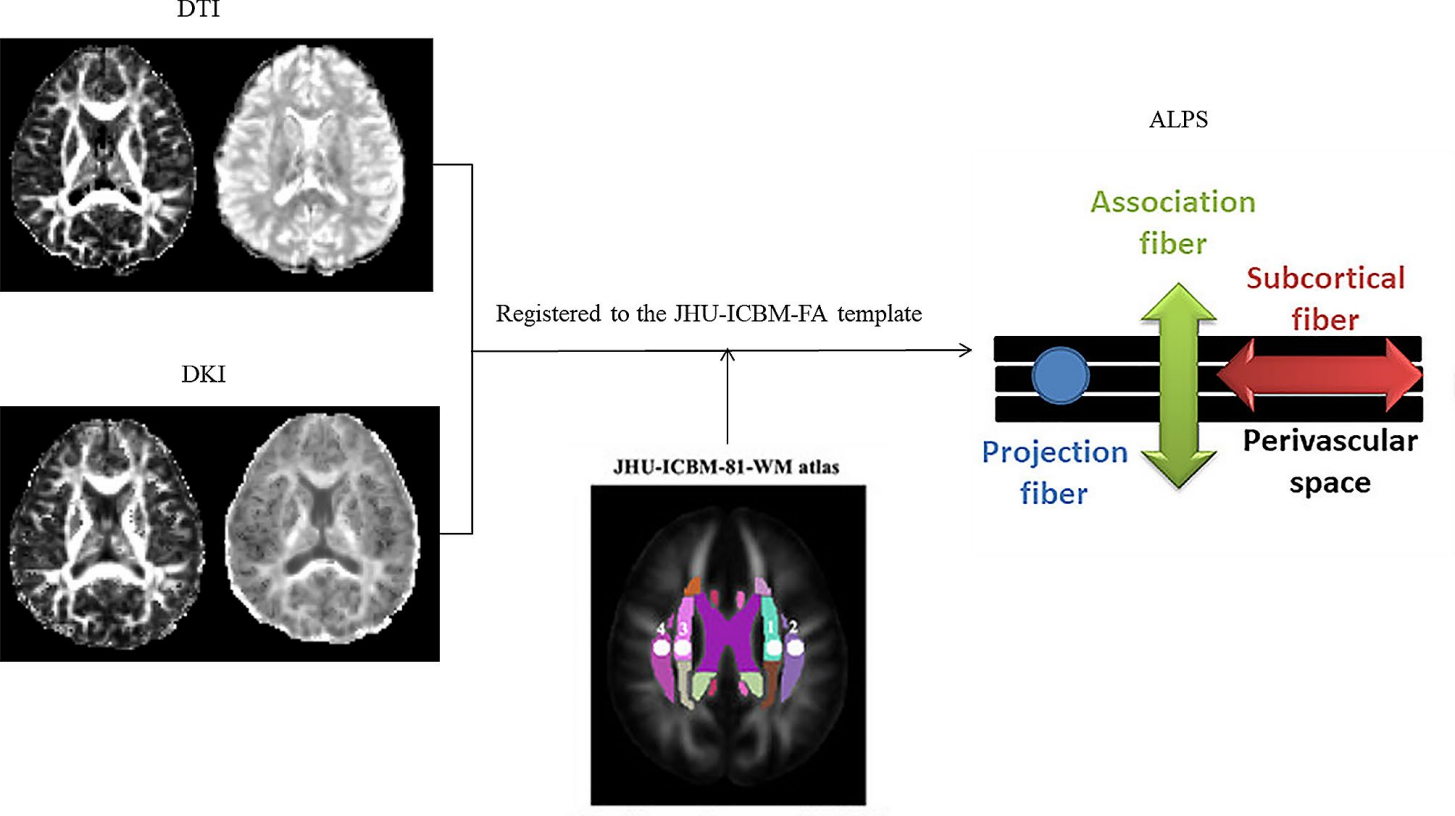
DTI-ALPS and DKI-ALPS indices calculation flowchart. Using the transformation matrix that registers the FA image to the standard space, we registered the diffusivities maps (Dxx, Dyy, Dzz, Kxxxx, Kyyyy, Kzzzz) to the standard space. Two ROIs were located on the projection fibre region (proj), where the main fibre extends along the z-axis, and two ROIs were located on the association fibre region (asso), where the main fibre extends along the y-axis. Finally, we extracted the diffusion diffusivities and kurtosis diffusivities of the 4 ROIs to calculate DTI-ALPS and DKI-ALPS, respectively. DTI-ALPS: diffusion tensor image analyses along with the perivascular; DKI-ALPS: diffusion kurtosis image analyses along with the perivascular

The DTI-ALPS and DKI-ALPS indices were calculated with a shared bash script (https://github.com/gbarisano/alps), which exhibited a high degree of reliability and repeatability (test-retest repeatability: ICC = 0.89 to 0.95, *P* < 0.001) [16]. We extracted the diffusion diffusivities of the diffusion tensor and kurtosis tensors along the x, y, and z axes. The diffusion tensor diffusivities are labelled Dxx, Dyy and Dzz respectively. The diffusivities of the kurtosis tensor are labelled Kxxxx, Kyyyy and Kzzzz, respectively. Using the transformation matrix that registers the FA image to standard space, we registered the diffusivity maps (Dxx, Dyy, Dzz, Kxxxx, Kyyyy, Kzzzz) to standard space. Four 5 mm diameter spherical regions of interest (ROI) were manually outlined on the JHU-ICBM-FA template. Two ROIs were located in the projection fibre region (proj), where the main fibre extends along the z-axis, and two ROIs were located in the association fibre region (asso), where the main fibre extends along the y-axis. Finally, we extracted the diffusion diffusivities and kurtosis diffusivities of the 4 ROIs to calculate the DTI-ALPS and DKI-ALPS indices, respectively (Fig. 2).

$$\begin{gathered} DTI - ALPS = mean\left( {Dxxproj,Dxxassoc} \right)/ \hfill \\\,\,\,\,\,\,\,\,\,\,\,\,\,\,\,\,\,\,\,\,\,\,\,\,\,\,\,\,\,\,\,\,\,\,\,\,mean\left( {Dyyproj,Dzzassoc} \right) \hfill \\ \end{gathered}$$

$$\begin{gathered} DKI - ALPS = mean\left( {Kxxxxproj,Kxxxxassoc} \right)/ \hfill \\\,\,\,\,\,\,\,\,\,\,\,\,\,\,\,\,\,\,\,\,\,\,\,\,\,\,\,\,\,\,\,\,\,\,mean\left( {Kyyyyproj,Kzzzzassoc} \right) \hfill \\ \end{gathered}$$

Where Dxxproj and Kxxxxproj are the diffusivities along the x-axis in the projection fibre, Dxxxxassoci and Kxxxxassoci are the diffusivities along the x-axis in the association fibre, Dyyproj and Kyyyyproj are the diffusivities along the y-axis in the projection fibre, and Dzzassoc and Kzzzzassoc are the diffusivities along the z-axis in the association fibre. We calculated the DTI-ALPS and DKI-ALPS indices for the left brain, the right brain and the whole brain.

### Statistical analysis

The Mann-Whitney U-test was used to compare the demographics of the two groups to determine whether or not there were significant differences. Chi-square was used to compare the gender composition of the two groups.

The ALPS index was tested with the Lilliefors test to see if it belonged to a normal distribution. A two-sample t-test was then used to compare the DTI-ALPS and DKI-ALPS indices of the two groups. We examined the correlation between the DTI-ALPS and DKI-ALPS indices and clinical variables. The statistical threshold was set at *p* < 0.05.

## Results

### Neuropsychological results

There were no significant differences in age (*z* = -4.777, *P* = 0.1774), gender distribution (*χ2* = 2, *P* = 0.1573), or years of education (*z* = 5.427, *P* = 0.1573) between the two groups. Details of the demographic data and associated tests are shown in Table 1.

**Table 1:** Demographics and neuropsychological data

	Patients with migraine	HC	z/χ^2^	*p*
Gender, n (M/F)	39(11/27)	29(17/12)	2	0.1573
Age, years	34.4 ± 9.7	25.6 ± 6.7	-4.777	0.1774
Education(years)	17 ± 3	13 ± 4	5.427	0.1573
Duration(years)	10.5 ± 9.5	-	-	-
Frequency(d/m)	3.0 ± 4.5	-	-	-
PHQ-6	1.59 ± 1.48	-	-	-
GAD-7	1.43 ± 1.71	-	-	-
MIDAS	13.4 ± 20.1	-	-	-
HIT-6	53.6 ± 15.9	-	--	-

Data represent mean ± SD. HC: Healthy controls; d/m: day per month; HIT-6: Headache Impact Test-6; MIDAS: Migraine Disability Assessment; PHQ-9: Patient Health Questionnaire-9; GAD-7: General Anxiety Disorder-7.

### Differences between the diffusion kurtosis image analyses along with the perivascular indices of the patients with migraine and healthy controls

**Fig. 3:**
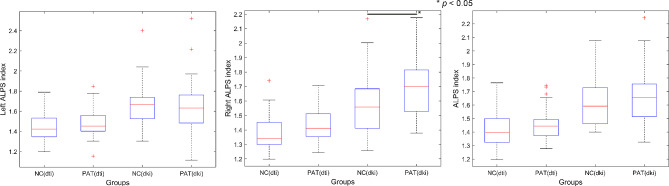
Statistical comparison of ALPS indices between migraine patients and healthy control groups. The right DKI-ALPS index in migraine patients was significantly higher than that in healthy controls (1.6858 vs. 1.5729; *p* = 0.0301). DKI-ALPS: diffusion kurtosis image analyses along with the perivascular

Compared to healthy controls, migraine patients showed increased DTI-ALPS and DKI-ALPS indices. However, only the right DKI-ALPS index was significantly increased (1.6858 vs. 1.5729; *p* = 0.0301) (Fig. 3; Table 2).

**Table 2:** DTI-ALPS and DKI-ALPS indices in the patients with migraine group compared with the healthy control group

	Left DTI-ALPS	Right DTI-ALPS	DTI-ALPS	Left DKI-ALPS	Right DKI-ALPS*	DKI-ALPS
Patients with migraine	1.48 ± 0.14	1.44 ± 0.12	1.46 ± 0.12	1.64 ± 0.26	1.69 ± 0.20	1.66 ± 0.20
HC	1.44 ± 0.15	1.38 ± 0.13	1.41 ± 0.13	1.65 ± 0.22	1.57 ± 0.21	1.62 ± 0.18

*P < 0.05. DTI-ALPS: diffusion tensor image analyses along with the perivascular; DKI-ALPS: diffusion kurtosis image analyses along with the perivascular; HC: healthy controls.

### Correlation analysis

There was no significant correlation between DTI-ALPS and DKI-ALPS indices and clinical variables.

## Discussion

In the current study, we proposed to use DKI to calculate the ALPS index. The right DKI-ALPS index of patients with migraine was significantly higher than that of healthy controls.

The DKI model was developed to solve the problem of nerve fibre crossing. As mentioned above, there are crossing fibres in 90% of the white matter of the brain [7, 17], so the diffusivity based on the diffusion tensor model is often biased. When it comes to describing the complex water diffusion processes in vivo, DKI provides a more comprehensive approach than DTI [18]. By estimating diffusivity and kurtosis, it can provide improved sensitivity and specificity in MR diffusion characterisation of neural tissue [18]. It is generally accepted that voxels with multiple fibre populations become less common as spatial resolution increases [19]. However, as spatial resolution increases, so does the frequency of crossing fibres. Rather than being a technical problem that can be solved by advances such as higher fields and stronger gradients, the crossing fibre problem appears to be a fundamental limitation of diffusion MRI related to the complexity of brain tissue [19]. Therefore, there is always a potential bias in the evaluation of glymphatic system function with the DTI-ALPS index, and the DKI-ALPS index should be used in the evaluation of glymphatic system function at the same time as the DTI-ALPS index.

Our study found no significant correlation between ALPS indices and clinical variables. Flow within the glymphatic system can be influenced by many factors, such as changes in arteriovenous hydrostatic pressure gradients, vasodilation or vasoconstriction, and intracranial pressure [2]. The influx of cerebrospinal fluid in the glymphatic system is inversely proportional to the heart rate, i.e., the faster the heart rate, the lower the glymphatic system function [20]. Hypertension can also inhibit the arterial pulses in the glymphatic system and reduce glymphatic system function [21]. In addition, the ALPS index is typically calculated on slices of the lateral ventricle body, representing only a portion of the total glymphatic system function as a marker of activity [2].

Patients with migraine showed increased DKI-ALPS index compared to healthy controls. Zhang et al. found that the right DTI-ALPS index in patients with CM was higher than that in healthy controls [5]. They hypothesized that the increased function of the glymphatic system in CM patients may be related to downstream vascular responses induced by CGRP release. However, DTI-ALPS results are inconsistent for glymphatic system function in patients with headaches. A study based on 92 healthy controls and 82 patients with migraine did not find a significant difference in the DTI-ALPS index between the two groups [6]. In addition, Kim et al.‘s study found that the DTI-ALPS index in patients with cluster headaches was lower than those in healthy controls [22]. The pathophysiology of cluster headache is somewhat similar to that of migraine, often associated with abnormal activity in the hypothalamus, trigeminal nerve, and autonomic nervous system [23]. Further studies should be conducted to confirm the underlying mechanisms of glymphatic system function in migraine patients by combining CGRP, inflammatory cytokines, or CSD exciting [2].

Our results showed that the right DKI-ALPS index in migraine patients was greater than in healthy controls. Studies using fMRI have also found a laterality of brain function in migraine patients [2, 24–26]. During acute painful stimulation, activation of the pain matrix is either confined to the right hemisphere or strongly biased to the right [24]. The visual stimulation study of fMRI revealed that healthy subjects showed activation of bilateral visual functional networks, while migraine patients showed activation of right-sided visual functional networks [25]. Functional MR imaging studies hypothesized that the predominance of right hemisphere dysfunction in migraine may arise from abnormal connections between the right thalamus and some ipsilateral cortical regions involved in pain regulation (primary somatosensory cortex and premotor cortex) [2, 26]. The validity of this hypothesis needs to be further investigated.

Several limitations should be considered when interpreting the current results. First, the DKI-ALPS method is an experimentally invalidated method and multi-site and large sample data are needed to validate the reliability and efficacy of this method. Secondly, this was a retrospective study. The study confirmed that changes in glymphatic system function were found in patients with migraine; however, it is not clear whether this change is the result or the cause of migraine. Thirdly, we only looked at glymphatic function in migraine patients, and migraine patients have different subtypes, such as migraine with aura, migraine with chronic migraine. The function of these subtypes of glymphatic system needs to be studied further.

## Conclusions

We proposed to use the DKI-ALPS index to evaluate glymphatic system function. There was a significant correlation between DKI-ALPS and DTI-ALPS indices. The right DKI-ALPS index in migraine patients was significantly higher than that in healthy controls. DKI-ALPS index is a potential indicator of glymphatic system function.

## Data Availability

No datasets were generated or analysed during the current study.
